# Supplementary material for: ARID1A promotes genomic stability through protecting telomere cohesion
Source: Nat Commun. 2019 Sep 6;10:4067. doi: 10.1038/s41467-019-12037-4 (PMC6731242; doi:10.1038/s41467-019-12037-4)
Supplement: Supplementary file 1 — Supplementary Information [file 41467_2019_12037_MOESM1_ESM.pdf]

ARID1A promotes genomic stability through protecting telomere cohesion

Zhao et al.

Supplementary Figures and Figure Legends

Supplementary Figure 1

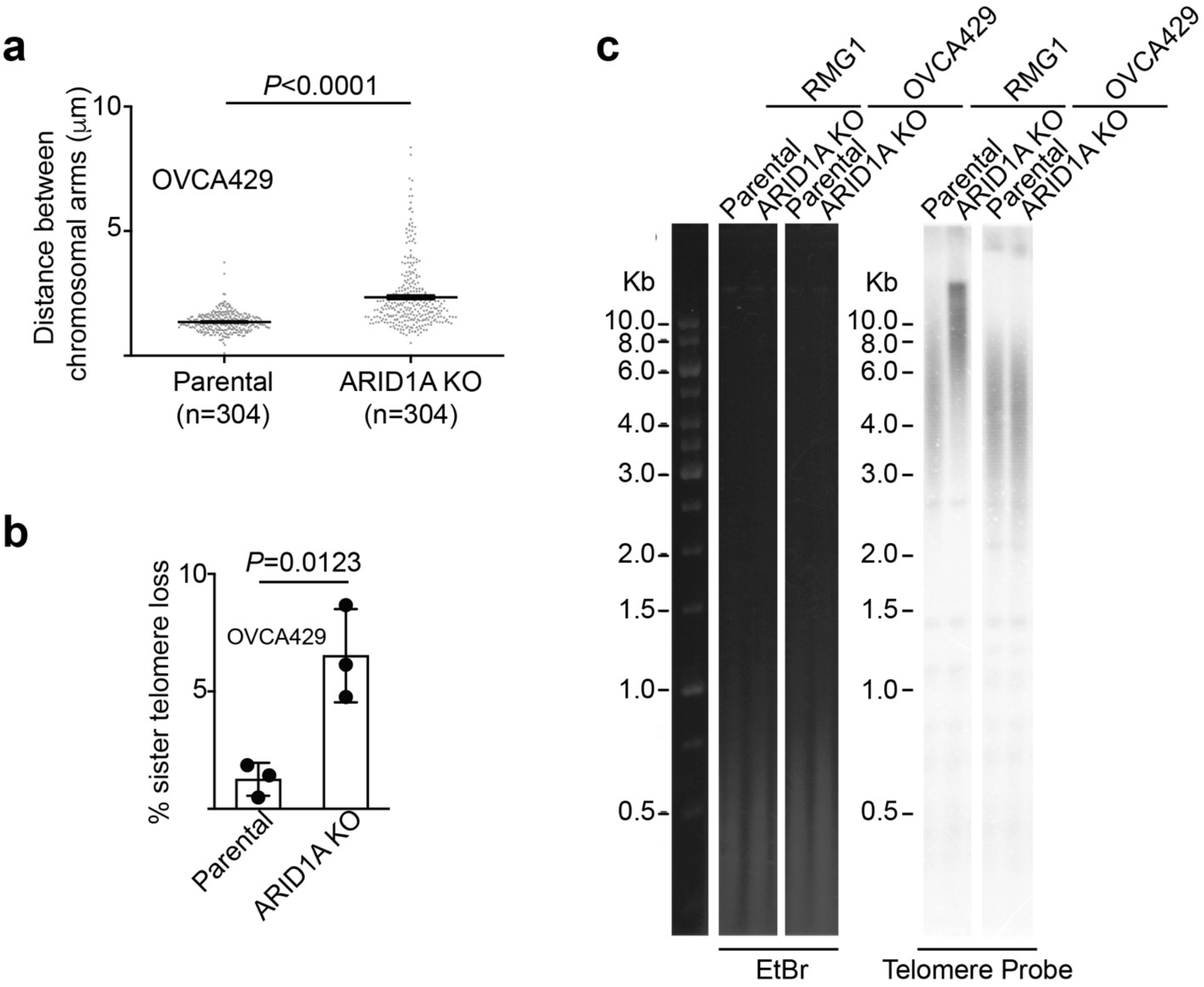

Supplementary Figure 1. ARID1A inactivation causes telomere cohesion defects
a, b, Quantification of distance between distal ends of sister chromatids (**a**) and percentage of cells with sister telomere loss (**b**) in parental and *ARID1A* knockout OVCA429 cells based on chromosome spread analysis. **c** telomere length analysis in parental and *ARID1A* knockout RMG1 and OVCA429 cells. n=3 independent experiments unless otherwise stated. Data represent mean ± s.e.m. *P* values were calculated using a two-tailed t-test.

Supplementary Figure 2

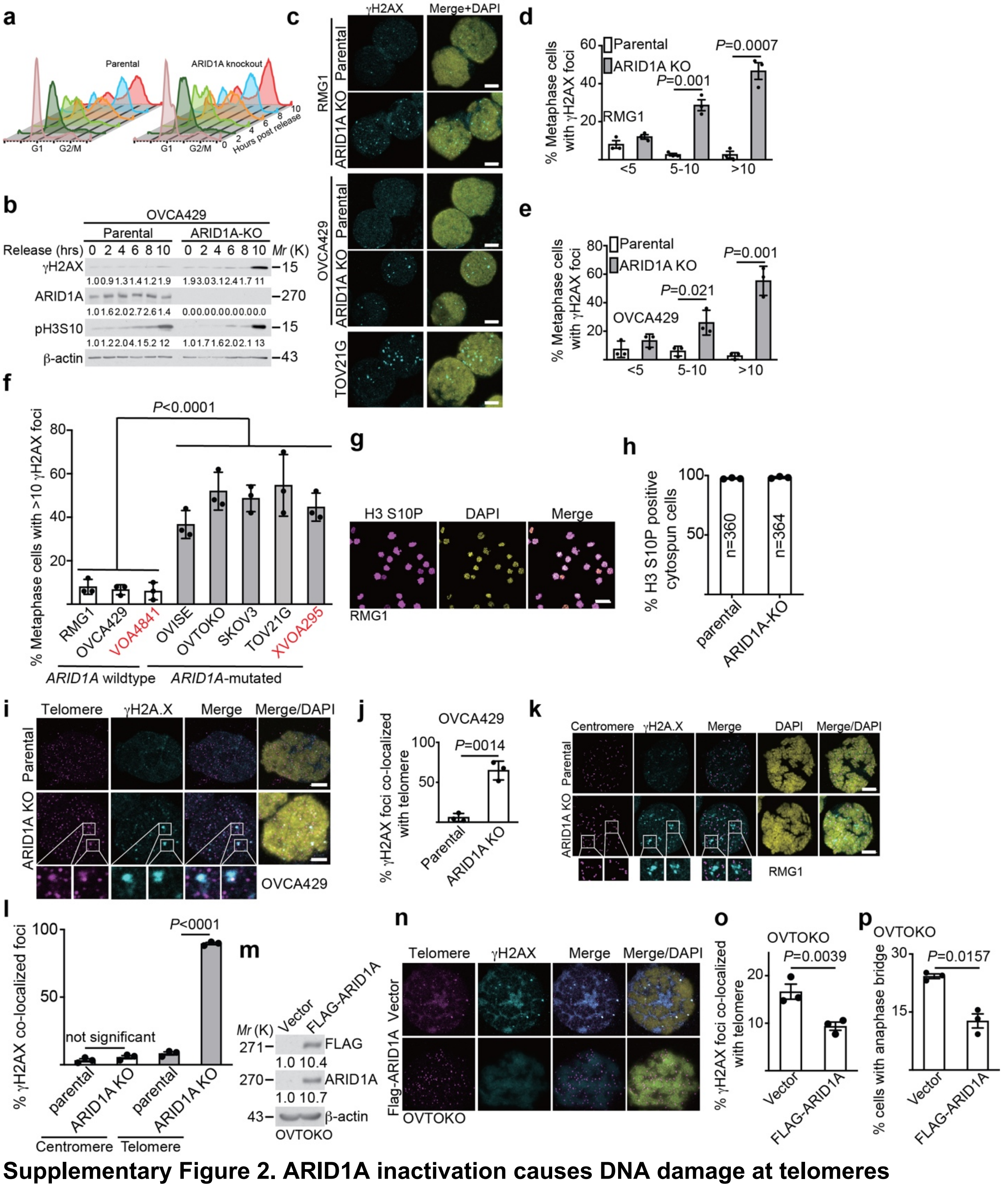

Supplementary Figure 2. ARID1A inactivation causes DNA damage at telomeres
a, Cell cycle profile of parental and ARID1A knockout RMG1 cells after release from double-thymidine synchronization at the indicated time points as determined by flow cytometry analysis. **b,** Immunoblot of DNA damage marker γH2AX in parental and ARID1A knockout OVCA429 cells. Phosphorylated Histone H3 at serine 10 (pH3S10) was used as a marker of mitosis. **c-e,** Representative images of γH2AX and DAPI staining of mitotic cells of the indicated cell lines (**c**) and quantification of mitotic γH2AX foci formation in parental and ARID1A knockout RMG1 (**d**)

and OVCA429 (**e**) cells. **f,** Quantification of γH2AX foci formation in metaphase cells in a panel of clear cell ovarian cell lines or primary cultures highlighted in red. **g, h**, Staining of cytospun cells with metaphase marker serine 10 phosphorylated histone H3 (H3 S10P) (**g**) and quantification of percentage of H3 S10P positive cells (**h**) in metaphase parental and *ARID1A* knockout RMG1 cells. **i, j**, Co-staining of telomere FISH and γH2AX (**i**) and quantification of percentage of cells with telomeric DNA damage (**j**) in metaphase parental and *ARID1A* knockout OVCA429 cells. **k**, **l**,Co-staining (**k**) and quantification (**l**) of centromere using anti-centromere antibodies protein (derived from human CREST patient serum) and γH2AX in metaphase parental and *ARID1A* knockout RMG1 cells. **m-p**, *ARID1A* mutant OVTOKO cells with or without re-expressing a FLAG-tagged wild-type ARID1A (**m**) were co-stained with telomere FISH and γH2AX (**n**) and quantification of percentage of γH2AX foci co-localized with telomere (**o**) or percentage of cells positive for anaphase bridges (**p**). n=3 independent experiments unless otherwise stated. Data represent mean ± s.e.m. Scale bar = 10μm. *P* values were calculated using a two-tailed t-test except for S2f by multilevel mixed-effects models. Relative intensities of immunoblot bands were quantified underneath.

Supplementary Figure 3

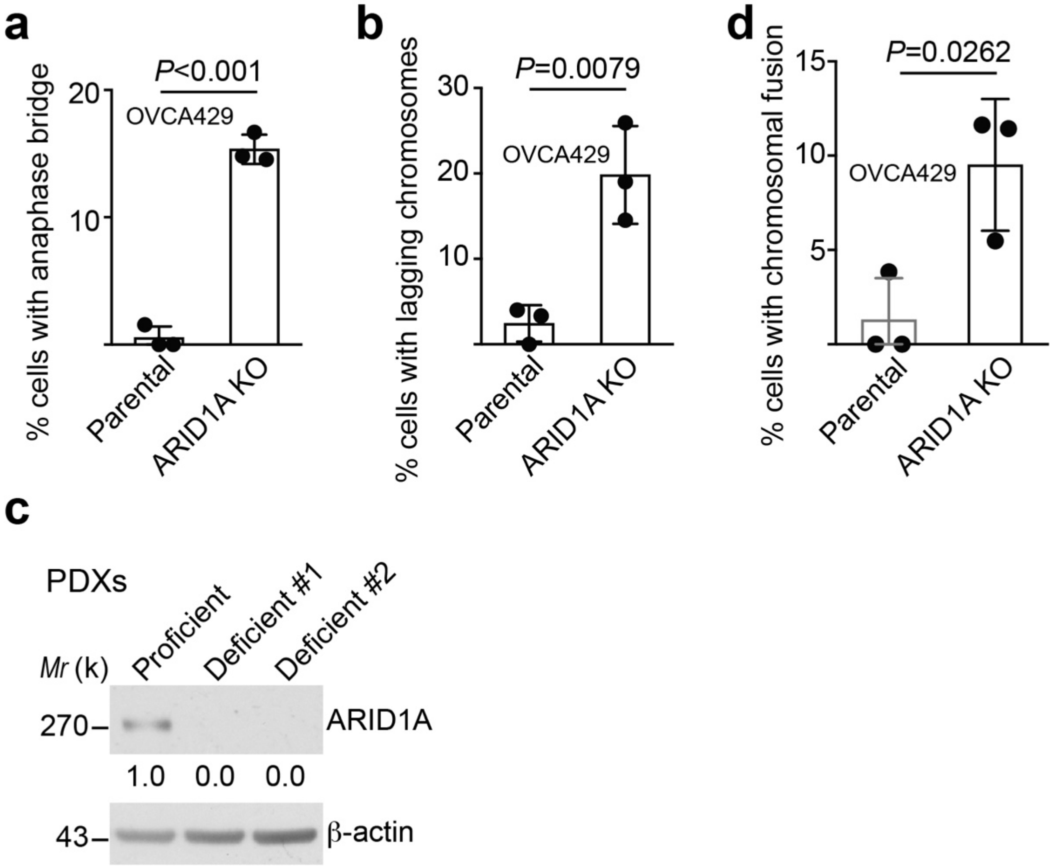

Supplementary Figure 3. ARID1A inactivation causes mitotic defects
a, b, Quantification of percentage of cells positive for anaphase bridge (**a**) and lagging chromosome (**b**) in mitotic parental and *ARID1A* knockout OVCA429 cells. **c,** ARID1A expression in the indicated patient derived xenografts of clear cell ovarian carcinomas determined by immunoblot. **d**, Quantification of percentage of cells positive for chromosome fusion in metaphase spread of parental and *ARID1A* knockout OVCA429 cells. n=3 independent experiments unless otherwise stated. Data represent mean ± s.e.m. *P* values were calculated using a two-tailed t-test. Relative intensities of immunoblot bands were quantified underneath.

Supplementary Figure 4

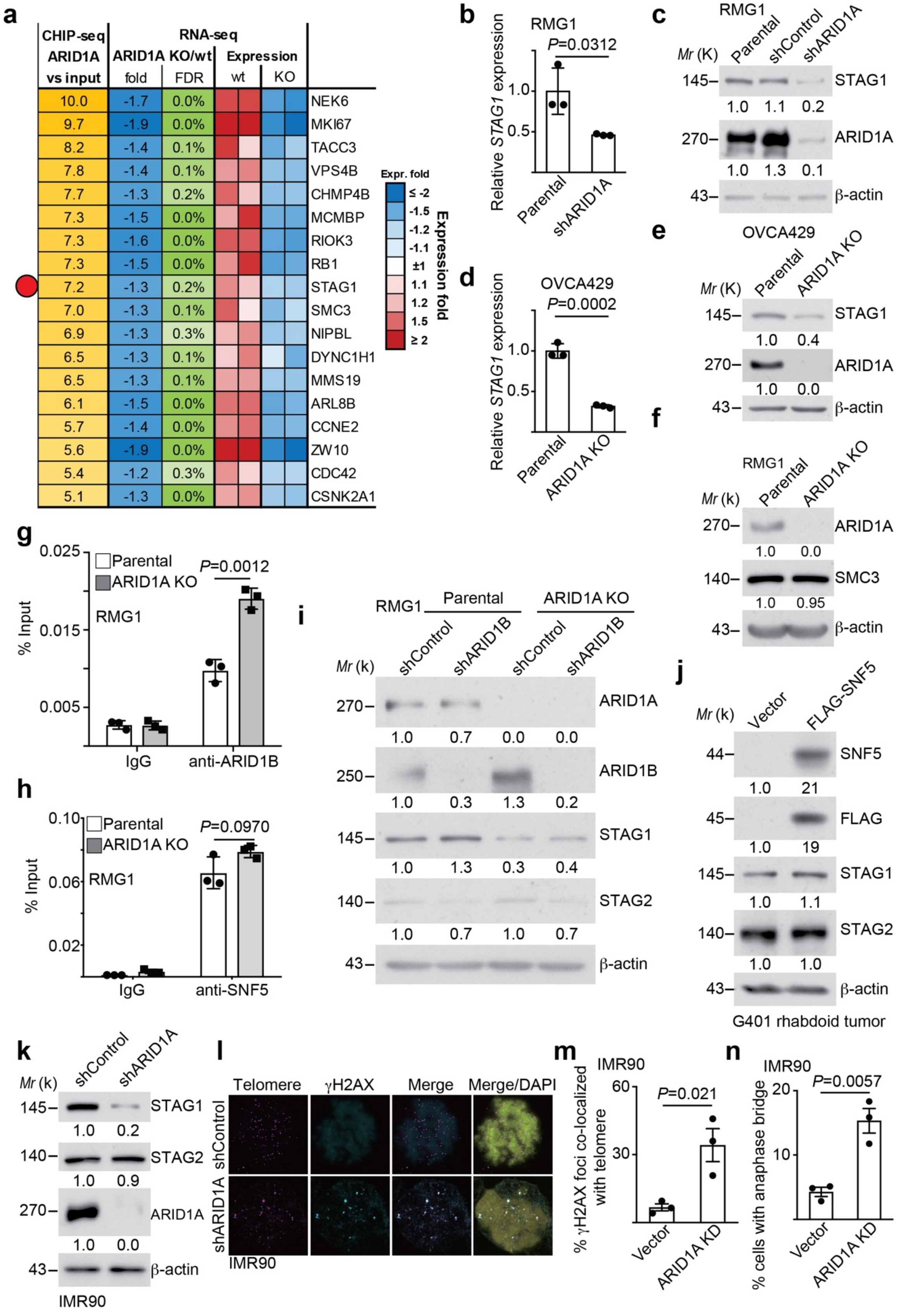

Supplementary Figure 4. *STAG1* is a direct ARID1A target gene
a, List of ARID1A target genes implicated in chromosome segregation function that includes genes implicated in sister chromatid cohesion as determined by cross-referencing ARID1A ChIP-seq and RNA-seq datasets of parental and ARID1A knockout RMG1 cells. Note that STAG1 is a direct ARID1A target gene that is known to regulate telomere cohesion and downregulated by ARID1A knockout. Two columns indicate two independent biological repeats. **b, c,** Downregulation of STAG1 at both mRNA levels determined by qRT-PCR (**b**) and protein levels determined by immunoblot (**c**) by shRNA mediated ARID1A knockdown in RMG1 cells. **d, e,** Downregulation of STAG1 at both mRNA levels determined by qRT-PCR (**d**) and protein levels determined by immunoblot (**e**) by ARID1A knockout in OVCA429 cells. **f,** Expression of cohesin subunit SMC3 and ARID1A in the indicated cells determined by immunoblot. **g,h,** ChIP analysis of ARID1B (**g**) and SNF5 (**h**) binding to the promoter of *STAG1* in parental and *ARID1A* knockout RMG1 cells. An isotype matched IgG was used as a negative control for ChIP analysis. **i,** Expression of ARID1A, ARID1B, STAG1 and STAG2 in ARID1A knockout RMG1 cells with or without shRNA mediated ARID1B knockdown. **j** Expression of SNF5, FLAG tag, STAG1 and STAG2 determined by immunoblot in SNF5 deficient G401 rhabdoid tumor cells with or without ectopic FLAG-SNF5 expression. k-n, Non-transformed primary human embryonic lung fibroblasts IMR90 cells with or without ARID1A knockdown were examined for STAG1 and STAG2 expression by immunoblot (**k**), co-stained with telomere FISH and γH2AX (**l**) and quantification of percentage of γH2AX foci co-localized with telomere (**m**) or percentage of cells positive for anaphase bridges (**n**). n=3 independent experiments unless otherwise stated. Data represent mean ± s.e.m. *P* values were calculated using a two-tailed t-test. Relative intensities of immunoblot bands were quantified underneath.

Supplementary Figure 5

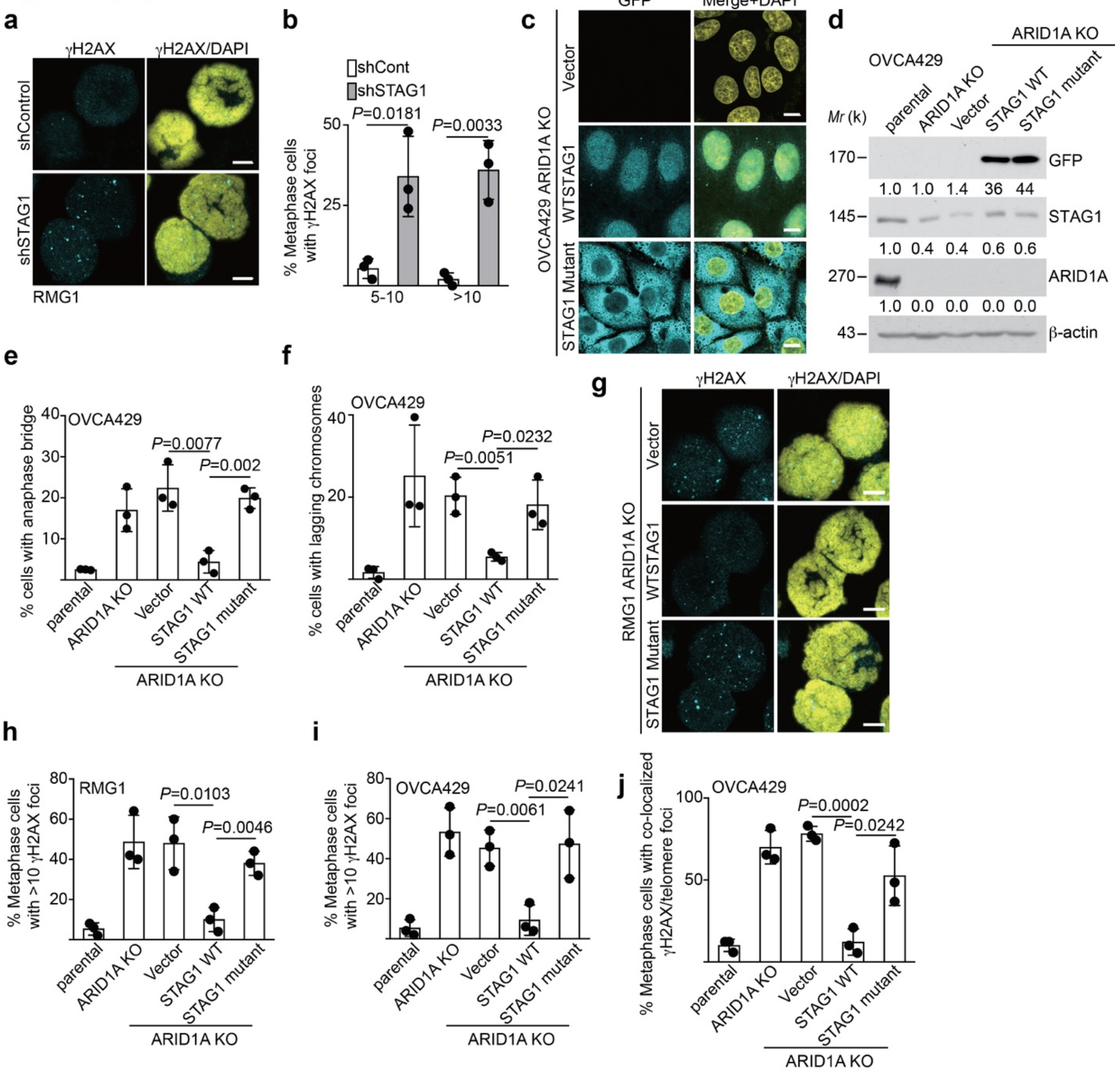

Supplementary Figure 5. Ectopic STAG1 expression rescues mitotic defects
a, b, Representative images of γH2AX staining in DAPI counter stained metaphase cells (**a**) and quantification of γH2AX foci formation in RMG1 cells expressing a control shRNA control or an shRNA against *STAG1* (**b**). **c, d,** Validation of ectopic GFP tagged STAG1 wild-type or mutant expression by immunofluorescence (**c**) or immunoblot (**d**) in *ARID1A* knockout OVCA429 cells. **e-f,** Percentage of anaphase bridge (**i**) and lagging chromosome (**j**) positive mitotic cells in the indicated parental, *ARID1A* knockout, and ARID1A knockout OVCA429 cells rescued with wild-type or mutant STAG1. **g,** Representative images of γH2AX staining in DAPI counter stained metaphase of the indicated RMG1 ARID1A knockout cells rescued with wild-type or mutant STAG1. **h-j,** Quantification of γH2AX foci formation in metaphase of the indicated RMG1 cells (**h**) and OVCA429 cells (**i**). In addition, percentage of telomere co-localized γH2AX foci positive parental, *ARID1A* knockout OVCA429 rescued with wild-type or mutant STAG1 was quantified (**j**). n=3 independent experiments unless otherwise stated. Data represent mean ± s.e.m. Scale

bar = 10μm. *P* values were calculated using a two-tailed t-test. Relative intensities of immunoblot bands were quantified underneath.

Supplementary Figure 6

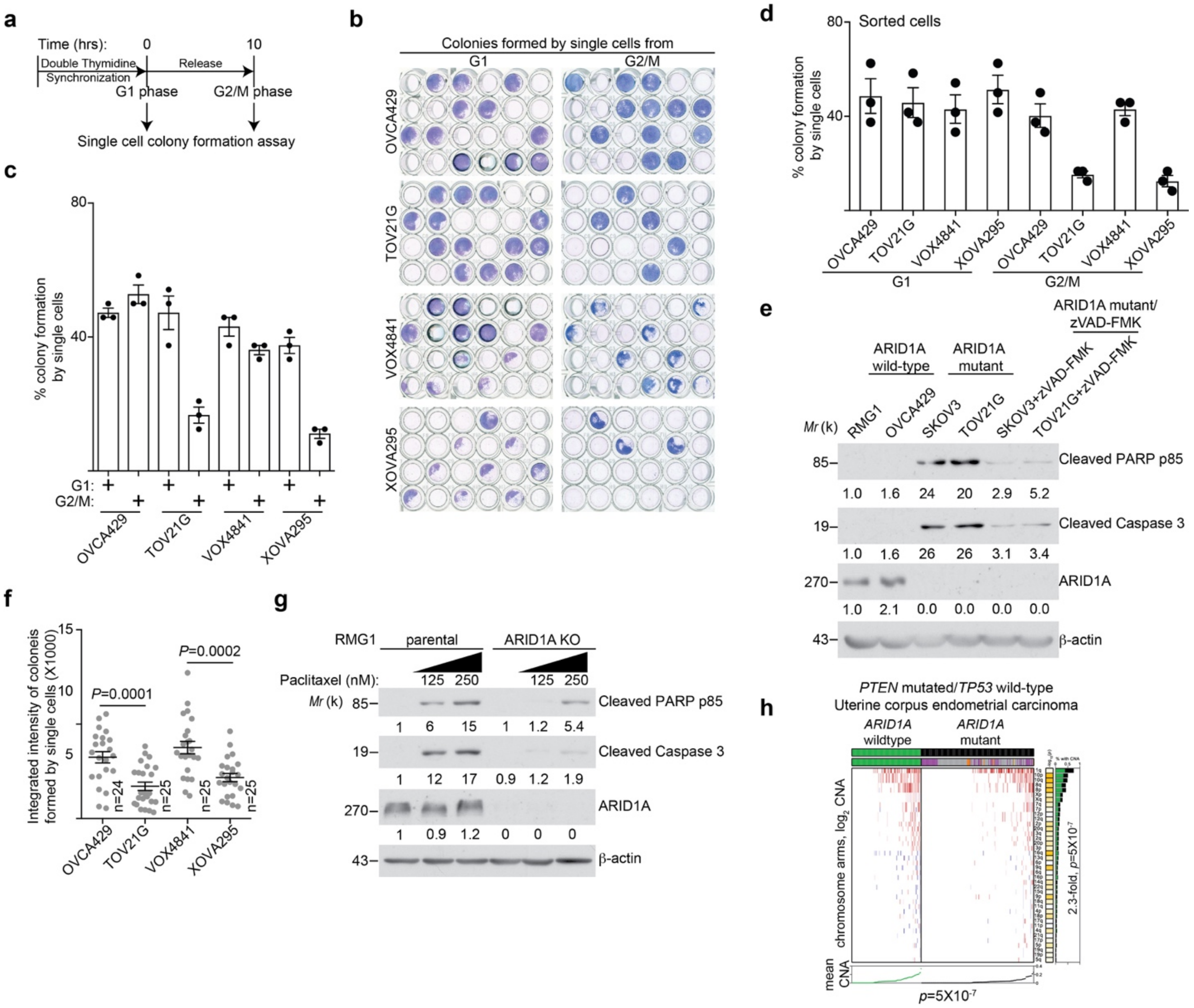

Supplementary Figure 6. ARID1A inactivation is selective against mitosis survival
a, Schematic of double thymidine synchronization and release to achieve G_1 phase and G_2/M phase cell cycle enriched cells for single cell colony formation assay. Please see Fig. S2a for cell cycle synchronization validation. **b, c,** Representative images (**b**) and quantification of colony formation efficiency (**c**) of colonies formed by single synchronized G_1 or G_2/M phases of the indicated cells. **d,** Quantification of colony formation efficiency of colonies formed by sorted G_1 or G_2/M phase single cells based on Hoechst 33342 staining of the indicated OCCC cells. **e,** Expression of ARID1A and apoptosis markers cleaved caspase 3 or cleaved PAPR p85 in the indicated *ARID1A*-mutated OCCC cell lines treated with or without a pan-Caspase inhibitor zVAD-FMK. **f**, Integrated density analysis performed by Image J of single cell colonies formed by G_1 phase of the indicated OCCC cells. **g,** The indicated *ARID1A* wild-type parental and ARID1A knockout RMG1 cells were treated with or without indicated concentrations of paclitaxel for 72 hours and examined for expression of markers of apoptosis such as cleaved caspase 3 and cleaved PARP p85 by immunoblot. **h**, In *PTEN* mutant and *TP53* wild-type uterine corpus endometrial carcinoma, *c*ompared with ARID1A wild-type tumors, ARID1A mutated tumors exhibit a significant less copy number variations in the TCGA datasets. n=3 independent experiments unless otherwise stated. Data represent mean ± s.e.m. *P* values were calculated using a two-tailed t-test. Relative intensities of immunoblot bands were quantified underneath.
