# Supplementary material for: ARID1A promotes genomic stability through protecting telomere cohesion
Source: Nat Commun. 2019 Sep 6;10:4067. doi: 10.1038/s41467-019-12037-4 (PMC6731242; doi:10.1038/s41467-019-12037-4)
Supplement: Supplementary file 3 — Description of Additional Supplementary Files [file 41467_2019_12037_MOESM3_ESM.pdf]

Description of Additional Supplementary Files

File Name: Supplementary Movie 1

Description: Live-cell time-lapse microscopy of ARID1A wild-type RMG1 parental (S1), ARID1A KO RMG1 (S2) or ARID1A-mutated TOV21G (S3) cells. An inverted wide-field fluorescent microscope Nikon Te300 was used under controlled CO2 (5%) and temperature (37 °C) for 24 hrs. The movie was analyzed and edited by NIS-elements AR software (Nikon).

File Name: Supplementary Movie 2

Description: Live-cell time-lapse microscopy of ARID1A wild-type RMG1 parental (S1), ARID1A KO RMG1 (S2) or ARID1A-mutated TOV21G (S3) cells. An inverted wide-field fluorescent microscope Nikon Te300 was used under controlled CO2 (5%) and temperature (37 °C) for 24 hrs. The movie was analyzed and edited by NIS-elements AR software (Nikon).

File Name: Supplementary Movie 3

Description: Live-cell time-lapse microscopy of ARID1A wild-type RMG1 parental (S1), ARID1A KO RMG1 (S2) or ARID1A-mutated TOV21G (S3) cells. An inverted wide-field fluorescent microscope Nikon Te300 was used under controlled CO2 (5%) and temperature (37 °C) for 24 hrs. The movie was analyzed and edited by NIS-elements AR software (Nikon).

File Name: Supplementary Movie 4

Description: Live-cell time-lapse microscopy of ARID1A wild-type RMG1 expressing shSTAG1 (S4), ARID1A KO RMG1 rescued with a wild-type (S5) or a nucleus-localization deficient STAG1 (S6). An inverted wide-field fluorescent microscope Nikon Te300 was used under controlled CO2 (5%) and temperature (37 °C) for 24 hrs. The movie was analyzed and edited by NIS-elements AR software (Nikon).

File Name: Supplementary Movie 5

Description: Live-cell time-lapse microscopy of ARID1A wild-type RMG1 expressing shSTAG1 (S4), ARID1A KO RMG1 rescued with a wild-type (S5) or a nucleus-localization deficient STAG1 (S6). An inverted wide-field fluorescent microscope Nikon Te300 was used under controlled CO2 (5%) and temperature (37 °C) for 24 hrs. The movie was analyzed and edited by NIS-elements AR software (Nikon).

File Name: Supplementary Movie 6

Description: Live-cell time-lapse microscopy of ARID1A wild-type RMG1 expressing shSTAG1 (S4), ARID1A KO RMG1 rescued with a wild-type (S5) or a nucleus-localization deficient STAG1 (S6). An inverted wide-field fluorescent microscope Nikon Te300 was used under controlled CO2 (5%) and temperature (37 °C) for 24 hrs. The movie was analyzed and edited by NIS-elements AR software (Nikon).
